# Complete mitochondrial genome sequence of a brackish water clam *Corbicula japonica* (Cyrenidae: Bivalvia) collected from an estuary of Gwangyang Bay in Korea

**DOI:** 10.1080/23802359.2022.2122742

**Published:** 2022-09-15

**Authors:** Kiyun Park, Ihn-Sil Kwak

**Affiliations:** a Fisheries Science Institute, Chonnam National University, Yeosu, Republic of Korea; b Department of Ocean Integrated Science, Chonnam National University, Yeosu, Republic of Korea

**Keywords:** Mitochondrial genome, Corbicula

## Abstract

The complete mitochondrial genome of the clam *Corbicula japonica* is 17,432 bp in length. The sequence consists of 13 protein-coding, 2 ribosomal RNAs, and 22 transfer RNA genes (GenBank accession no. MZ895053). The proportion of base-pairs in *C. japonica* are A + T (70.5%) and G + C (29.5%). Phylogenetic analysis reveal *C. japonica* to be sister species to *C. fluminea* within the monophyletic genus *Corbicula*, with high support. This study is helpful to the classification of the brackish water clam *C. japonica*, which is difficult to identify during early development owing to variation of shell morphology.

The brackish water clam *Corbicula japonica* Prime, 1864, a filter-feeding bivalve, is widely distributed in rivers and brackish waters throughout the Asian region, and it is a commercially important species for food and inland fishery resource in Korea (Park et al. 2004; Koyama et al. 2015; Xie et al. 2018). Aquatic *C. japonica* clams inhabiting brackish waters are generally exposed to salinity changes and can accumulate water contaminants under highly stressful environments (Koyama et al. 2015; Zhang et al. 2020). The clams, such as *C. japonica* as well as *Corbicula fluminea,* have been proposed as a good bio-indicator for the assessment of aquatic environments, owing to their rapid growth and bioaccumulation (Koyama et al. 2015; Bertucci et al. 2018; Domingues et al. 2020). However, the classification of Asian *Corbicula* has been greatly complicated by the extraordinary morphological variation in shell characteristics (Park et al. 2004). Mitochondrial DNA (mtDNA) sequences such as those of the cytochrome c oxidase subunit I (COI) gene have been used to elucidate the phylogeny of Cyrenidae (Haponski and Foighil 2019). However, there are only limited number of completed mtDNA sequences in the Cyrenidae family (Wu et al. 2019; Zhang et al. 2019). In the present study, we determined the complete mitochondrial genome sequence of *C. japonica* collected from an estuary using Illumina next-generation sequencing. Specimens of *C. japonica* were collected from the estuarine area of Gwangyang Bay, Yeosu, South Korea (N 34°58.45′, E 127°46.02′) on September 2019. The article follows the ARRIVE guidelines (https://arrive-guidelines.org/). Genomic DNA was extracted from *C. japonica* whole body using the DNeasy blood & tissue kit (Qiagen, Valencia, CA, USA). The voucher specimen (CNUISI-021020130) was deposited at the Specimen Museum of Fisheries Science Institute, Chonnam National University (KY Park, ecoblue8@gmail.com). DNA sequencing using Illumina HiSeq4000 was carried out in Macrogen Inc., (Seoul, Korea). De novo assembly of cleaned reads was performed by various k-mer using SPAdes v.3.13.0 (Bankevich et al. 2012). After assembly, MitoZ (v.2.3), a Python3-based toolkit, was used for annotation (Meng et al. 2019).

The mitochondrial genome sequence of *C. japonica* is available at the National Center for Biotechnology Information (NCBI) database (GenBank accession number MZ895053). The complete sequence of the mtDNA of *C. japonica* is 17,432 bp and comprises 37 genes, including 13 protein-coding genes (PCGs), 22 tRNAs, and 2 rRNAs. All genes located on the same positive strand, compared with the majority of bivalves. The A + T base content (70.5%) was higher than the G + C content (29.5%). Nucleotide base composition is as follows: A (27.5%), C (9%), G (20.5%), and T (43%). The gene order is same with *C. fluminea*. A total of 22 tRNA sequences were found throughout the mitogenome of *C. japonica*, which identified based on their respective anticodons and secondary structures, ranged in length from 62 (*trnR, trnH, trnW*) to 68 bp (*trnM*). In the two rRNAs, *12 s rRNA* (rrnS) gene is 868 bp in length, which lies between the *trnT* and *trnM* genes, whereas *16 s rRNA* (rrnL) gene is 1236 bp in length lying between the *cob* and *atp8* genes. The start codon of *ATP8*, *ND2*, *ND6* was ATG, that of *COX1*, *COX2*, *CYTB*, *ND3* was ATT, that of *ND4*, *ND4L* was ATA, that of *ATP6*, *ND1* was GTG, and that of *ND5*, *COX3* was TTG. Two types of terminal codons (*TAA* and *TAG*) are used in all genes of PCGs.

To analyze the phylogenetic relationships of *C. japonica* and other 17 mitochondrial genomes from Cyrenoidea, Veneroidea, and Mactroidea superfamilies in Venerida, phylogenetic trees were obtained using maximum-likelihood analysis based on all gene sequences by MEGA-X software (Kumar et al. 2018) (Figure 1). The result of phylogenetic analysis showed that *C. japonica* formed a well-supported clade with *C. fluminea*, *C. similis*, and *C. leana* in Cyrenidae. *C. japonica* emerges as sister species to *C. fluminea.*

**Figure 1.**
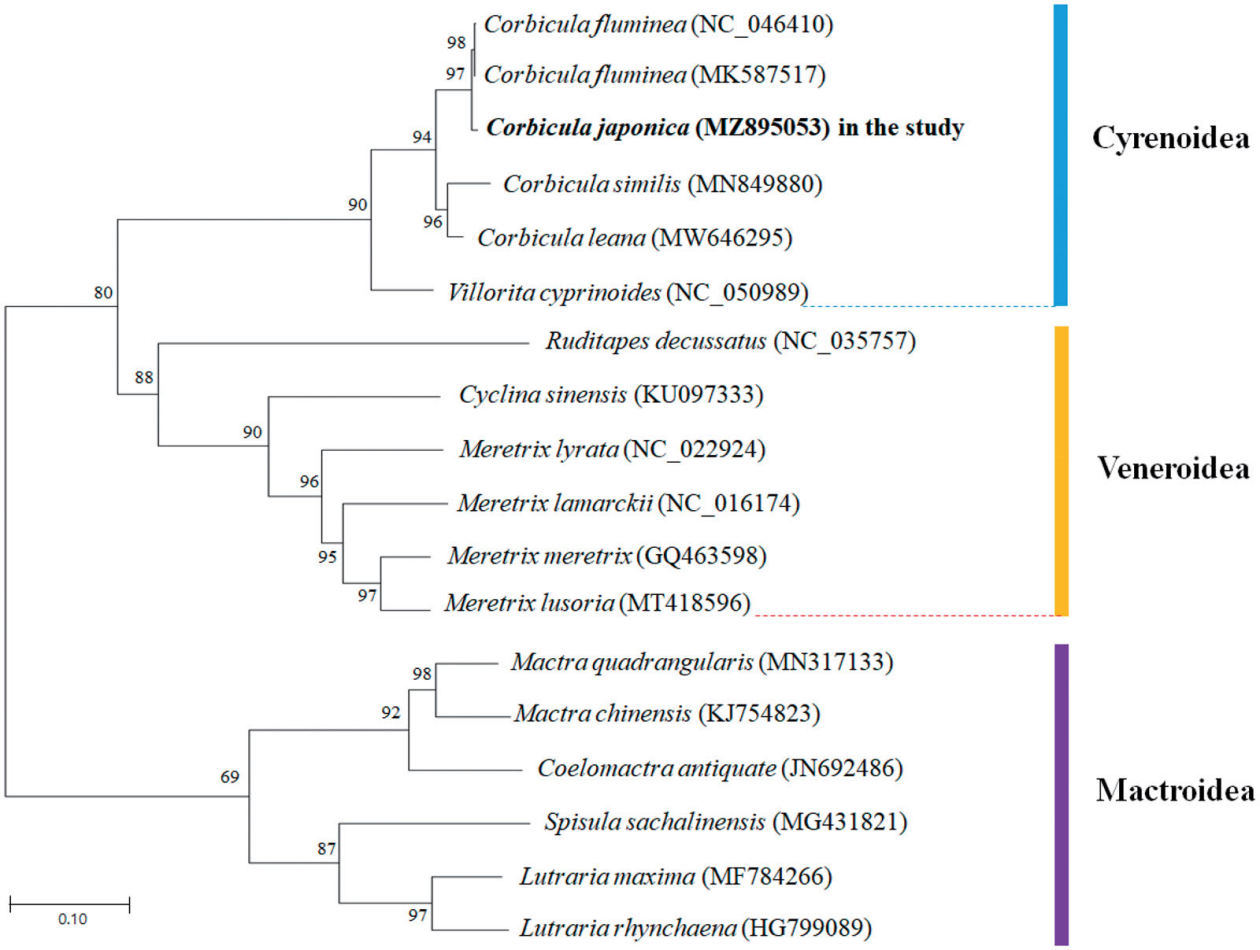
Phylogenetic tree constructed by maximum-likelihood method based on 18 Venerida complete mitochondrial genomes. All the bootstrap values after 1000 iterations are indicated at the nodes. The color bar presented the family.

## Ethical approval

Experiments were performed in accordance with the guidelines and regulations of the Animal Care and Use Committee of Chonnam National University (Yeosu, South Korea). This study did not involve Endangered or protected species.

## Author contributions

KP and ISK contributed to the conception and design of the study, analysis, and interpretation of the data. KP wrote the first version of the manuscript. KP and ISK critically reviewed the article regarding its intellectual content. KP and ISK collected biological samples. All authors read, discussed, and approved the final version and all authors agree to be accountable for all aspects of the work.

## Data Availability

The genome sequence data that support the findings of this study are openly available in GenBank of NCBI at (https://www.ncbi.nlm.nih.gov/) under the accession no. MZ895053. The associated BioProject, SRA, and Bio-Sample numbers are PRJNA803933, SRR17952277 and SAMN25690507, respectively.
